# White Matter Plasticity in the Adult Brain

**DOI:** 10.1016/j.neuron.2017.11.026

**Published:** 2017-12-20

**Authors:** Cassandra Sampaio-Baptista, Heidi Johansen-Berg

**Affiliations:** 1 Oxford Centre for Functional MRI of the Brain, Nuffield Department of Clinical Neurosciences, John Radcliffe Hospital, University of Oxford, Oxford OX3 9DU, UK

**Keywords:** white matter, plasticity, MRI, learning, myelin

## Abstract

The study of brain plasticity has tended to focus on the synapse, where well-described activity-dependent mechanisms are known to play a key role in learning and memory. However, it is becoming increasingly clear that plasticity occurs beyond the synapse. This review focuses on the emerging concept of white matter plasticity. For example, there is growing evidence, both from animal studies and from human neuroimaging, that activity-dependent regulation of myelin may play a role in learning. This previously overlooked phenomenon may provide a complementary but powerful route through which experience shapes the brain.

## Teaser

Sampaio-Baptista and Johansen-Berg review the current evidence supporting the important and previously neglected role of white matter plasticity in learning and experience during adulthood.

## Main Text

### Introduction

White matter (WM), consisting of axons connecting different brain regions, constitutes about half of the total human brain volume. Axons can be myelinated or unmyelinated, and it is myelin’s chemical composition of mainly lipids that gives the WM its characteristic color. If the myelinated axons of an adult human brain were laid out end to end, the total length would reach approximately 160,000 km (Marner et al., 2003).

Besides axons, WM also contains myelin-producing oligodendrocytes, astrocytes, microglia, and oligodendrocyte precursor cells (OPCs). In the CNS of the rat, each oligodendrocyte can myelinate more than 20 axons, but this varies between brain areas (Chong et al., 2012, Davison and Peters, 1970). Myelin is formed in segments with the unmyelinated axonal areas between segments called nodes of Ranvier. The primary function of myelination is to speed conduction of the electric impulse along an axon, allowing the action potential to travel long distances faster (reviewed in Freeman et al., 2016, Hartline and Colman, 2007). Other features of WM such as axon diameter, internode length, ion channel density, and myelin thickness also affect conduction speed. In gray matter, myelin plays a role in neurite growth inhibition (McKerracher et al., 1994), potentially limiting plasticity once a circuit has been formed (McGee et al., 2005). Complex cortical networks tend to be less myelinated (McGee et al., 2005) and thus more prone to plasticity mechanisms, suggesting that myelin might provide the finishing touches in circuit formation.

Myelination is so important for the proper function of the nervous system that it is thought to have evolved independently in several distinct animal branches (Hartline and Colman, 2007). Myelination is particularly important in larger brains, where finely calibrated conduction speeds are necessary for signal coordination across long distances. Brain tracts like the corpus callosum are not fully myelinated and there is great variation in myelination from brain region to region (Sturrock, 1980, Young et al., 2013). Furthermore, axons can exhibit long unmyelinated stretches between myelinated segments (Tomassy et al., 2014) as well as variation in internode length (Ford et al., 2015). This suggests that faster is not always better; instead, myelination is finely and locally tuned in order to coordinate the timing of action potentials that may require both high and low conduction speeds (Waxman, 1997). Recent simulations support this view (Arancibia-Cárcamo et al., 2017, Etxeberria et al., 2016, Ford et al., 2015).

The importance of WM to brain health has long been appreciated due to devastating effects of WM diseases such as multiple sclerosis. However, WM has traditionally taken a backseat role in our understanding of behavior and is typically considered to simply provide a route for communication between neurons. Similarly, conceptions of how brain connections change with experience have naturally focused on the synapse, where Hebbian plasticity mechanisms such as long-term potentiation provide a powerful substrate for learning. However, it is becoming increasingly clear that WM plasticity offers a complementary route through which experience can shape brain connections. Recent evidence from both human (Scholz et al., 2009) and rodent neuroimaging studies (Blumenfeld-Katzir et al., 2011, Sampaio-Baptista et al., 2013), as well as knockout mouse models (McKenzie et al., 2014), shows that WM demonstrates dynamic, experience-dependent plasticity that contributes to learning in the adult brain.

In the next sections, we will discuss possible cellular mechanisms of WM plasticity, such as new myelin formation, changes in myelin thickness, internode length modulation, and alterations in the nodes of Ranvier. We will also summarize activity-dependent myelination mechanisms that might underlie experience-dependent changes in the adult brain. Finally, we will review the current evidence from human and animal models that provides support for WM plasticity in response to learning and experience during adulthood.

### Cellular Mechanisms of White Matter Plasticity

Experience-dependent WM plasticity requires mechanisms through which activity along an axon can alter the structural properties of that axon. For this process to be relevant to learning, these alterations in structure would in turn be associated with changes in the functional properties of the axon, giving rise to alterations in behavior. As discussed above, alterations in the structural properties of the axon, such as myelin, axon diameter, or internode length, give rise to changes in physiological properties such as conduction speed, which will have relevance to behavior (Fields, 2015).

To date, most work on WM plasticity has focused on activity-dependent changes in myelination, though changes in other properties, such as nodes of Ranvier or internode length, may potentially play a role (Ford et al., 2015). Alterations in myelin may occur through changes in myelin thickness by pre-existing oligodendrocytes, or by new myelin formation through differentiation of OPCs into new oligodendrocytes.

#### Oligodendrocyte Precursor Cells in the Adult Brain

**Figure 1:**
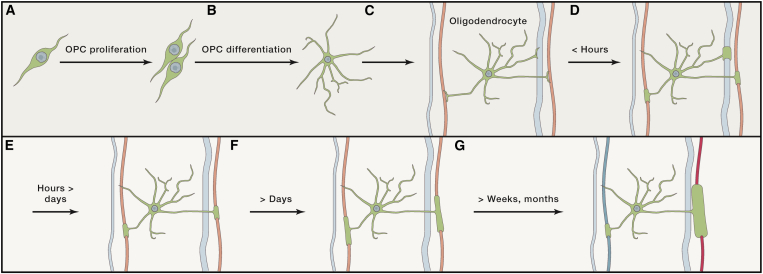
Schematic of Sequence of Events in Myelin Formation and Remodeling (A–C) OPCs (A) proliferate then (B) differentiate into oligodendrocytes, which (C) establish axonal segments. (D) The number of myelin segments is established within a few hours. Axon size and activity influence which axons are myelinated (active axons indicated in light red). (E) Occasional retractions can occur after this period. (F) Myelin sheaths are wrapped around the selected axons. (G) Axonal activity potentially modulates myelin thickness and length after this period (active axon in red and inactive in blue).

**Figure 2:**
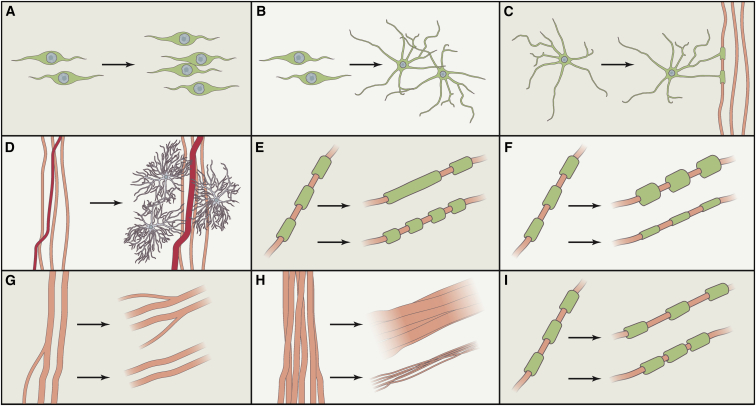
Summary of Possible Activity or Learning-Dependent Structural Changes that May Occur in White Matter during Adulthood (A) OPC proliferation. (B) OPC differentiation. (C) New myelination by adult-born oligodendrocytes. (D) Vascular and glial changes. (E) Changes in the internode length. (F) Myelin remodeling (increases or decreases in thickness) by preexisting oligodendrocytes. (G) Axonal branching or pruning (not yet demonstrated in WM in response to experience). (H) Axon diameter increases or decreases. (I) Changes in nodes of Ranvier length.

OPCs are a subtype of glial cells that have the capacity to differentiate into myelin-forming oligodendrocytes (Figures 1 and 2A–2C). During the postnatal period, many OPCs differentiate and develop into oligodendrocytes (Kessaris et al., 2008), but a significant number remain as OPCs into adulthood. It is estimated that around 5% of the total cells in the adult rat brain are OPCs (Dawson et al., 2003). NG2-positive cells have also been identified in the human brain (Chang et al., 2000), with suggestions that OPCs might represent 10%–15% of total human glial cells (Staugaitis and Trapp, 2009). These OPCs have the potential to proliferate, differentiate, and form new myelinating oligodendrocytes in adulthood (Hughes et al., 2013, Young et al., 2013) (Figures 1 and 2A–2C). Indeed, rodent studies show that more than 20% of myelinating oligodendrocytes present in the adult brain are adult-born (Rivers et al., 2008).

A subclass of OPCs can fire electric impulses. Although it is not clear what function is served by these spikes, it has been proposed that they provide a cell-wide signal that can be used to detect active axons and guide myelination (Fields, 2008, Káradóttir et al., 2008). It has also been demonstrated that OPCs extend their cellular processes to synapses (Bergles et al., 2000) in gray matter and to nodes of Ranvier in WM (Butt et al., 1999, Serwanski et al., 2017), suggesting OPCs might monitor action potentials at these sites.

#### Forming New Myelin during Adulthood

Recently matured oligodendrocytes establish all their myelin segments within a few hours in *in vitro* and *in vivo* zebrafish models (Czopka et al., 2013, Watkins et al., 2008). Although this suggests a short time window for segment formation, there is evidence that occasional segment retractions can occur after this period (Czopka et al., 2013) and that myelin thickness within each segment can be altered. For instance, in response to conditional upregulation of a cellular signaling pathway, preexisting oligodendrocytes of adult mice globally increased myelin thickness, resulting in faster nerve conduction velocity and enhanced hippocampal-dependent emotional learning (Jeffries et al., 2016). Although it is unclear why a global myelin change should be associated with such a specific behavioral improvement (no changes in motor learning or object recognition were detected), this finding provides evidence that preexisting oligodendrocytes in the adult brain retain the ability to regulate myelin thickness (Figure 2F). Additionally, mechanisms have been identified by which myelin thickness changes can be achieved by preexisting oligodendrocytes (Flores et al., 2008, Snaidero et al., 2014). In fact, it has been suggested that the majority of myelin remodeling occurring in the adult human brain is carried out by preexisting oligodendrocytes: ^14^C analysis of human brain revealed only a small number of new oligodendrocytes a few years after birth, but on the other hand, new and contemporary myelin could be detected. This finding could be related to renewal of myelin sheaths or to protein exchange in stable myelin sheaths (Yeung et al., 2014). This suggests that preexisting oligodendrocytes continually form new myelin and that new adult-born oligodendrocytes are less common in the human brain in comparison to the findings that 20% of the oligodendrocytes present in the adult rodent brain are adult-born (Rivers et al., 2008). It is not clear if the different conclusions of these two studies stem from a difference in species (humans versus nonhuman species) or due to the cell labeling methods employed.

Overall, there is accumulating evidence that newly formed oligodendrocytes and preexisting oligodendrocytes remodel myelin in the adult brain. This can be potentially altered by experience, and myelin thickness changes have been reported in relation to social isolation or neuronal stimulation studies (Gibson et al., 2014, Liu et al., 2012) (Figure 1F). However, it is not known if this is due to myelin remodeling by preexisting oligodendrocytes or if newly formed oligodendrocytes simply form thicker myelin in response to experience.

#### Activity-Dependent Modulation of Myelination

Which signals guide myelin modulation, and do any of these guiding signals support the idea of activity-dependent modulation of myelin (Figure 1)? Oligodendrocytes do not wrap astrocytes or dendrites (Althaus et al., 1984, Zalc and Fields, 2000), as dendrites inhibit myelin-guidance adhesion molecules, resulting in lack of myelination in these cell regions (Redmond et al., 2016). Fiber caliber is one determinant of myelination: oligodendrocytes can wrap around carbon fibers larger than 0.4 μm in the absence of electrical activity or axonal signaling, but myelination in such cases has previously been found to be abnormal and not well compacted (Althaus et al., 1984, Lubetzki et al., 1993). However, recent evidence suggests that oligodendrocytes *can* form compact myelin sheaths even in the absence of molecular axonal cues, and furthermore that sheath length depends not on properties of the fiber but on the regional origin of the oligodendrocyte (brain versus spinal cord) (Bechler et al., 2015).

Still, it has been proposed that myelination is modulated at least in part by axonal activity (Demerens et al., 1996, Ishibashi et al., 2006, Mensch et al., 2015, Stevens et al., 1998, Stevens et al., 2002, Wake et al., 2011). Most evidence of activity-dependent regulation of myelination comes from developmental studies. For instance, *in vitro* studies demonstrated that increases in neuronal activity, either by high-frequency stimulation or pharmacological manipulation, result in increased myelin sheath formation and myelin compaction within 2–14 days (Demerens et al., 1996, Ishibashi et al., 2006, Stevens et al., 1998), while low-frequency stimulation inhibits myelination compared to no stimulation (Stevens et al., 1998). A recent *in vivo* developmental study in zebrafish shed new light on mechanisms that direct myelination to specific axons, establishing that neuronal activity is important both for the appropriate selection of specific axons for wrapping and for myelin stabilization (Hines et al., 2015) (Figure 1). While oligodendrocytes initiated wrapping of large caliber axons even in the absence of neuronal activity, resulting myelin sheaths were only *maintained* on active axons (Hines et al., 2015) (Figures 1D–1G). In these studies, a modest increase in OPC number was also seen with increased activity (Hines et al., 2015). The above developmental studies suggest that myelination can be bidirectionally altered by neuronal activity during development.

To what extent may similar mechanisms persist into adulthood? A recent study reported that optogenetic stimulation of secondary motor cortex of adult mice increases OPC proliferation within 3 hr and results in increased myelin thickness and behavioral effects 4 weeks later (Gibson et al., 2014). These effects were not seen in a control group that did not express channelrhodopsin but received the same type of light stimulation. Controlling for light stimulation is important, as it has been demonstrated that stimulation alone, even in the absence of the light-sensitive channels, results in increased blood flow (Rungta et al., 2017). However, further evidence and replication are needed to effectively demonstrate a direct relationship between neuronal activity and myelination during adulthood.

The majority of studies in this area have assessed whether increasing activity results in increased myelination. Whether activity-dependent modulation of myelin can be *bidirectional*, such that reductions in activity result in reductions in myelin, remains an open question. The developmental zebrafish studies discussed above demonstrate that, in the absence of activity, new myelin is not maintained (Hines et al., 2015). During development, sensory deprivation leads to shortened myelin sheathes, resulting in reduced impulse conduction (Etxeberria et al., 2016). There are a few rodent studies of social isolation, which could theoretically be associated with lower neuronal activity, that indirectly suggest that lower brain activity results in lower myelination during both development (Makinodan et al., 2012) and adulthood (Liu et al., 2012).

In conclusion, current evidence suggests that oligodendrocytes are intrinsically programmed to myelinate large-caliber fibers (>0.4 μm) with inhibitory molecules preventing indiscriminate myelination of dendrites, while the regional origin of the oligodendrocytes influences the length of the internodes. On the other hand, external cues such as axon size and axonal activity can additionally regulate sheath length, number, and thickness, providing a mechanism by which the environment and behavior can fine-tune myelination (Figures 1 and 2). While most studies demonstrating neuronal activity-dependent regulation of myelin have been carried out in developmental models, at least one study has provided evidence of similar mechanisms occurring during adulthood (Gibson et al., 2014).

#### Axonal Sprouting and Changes in Nodes of Ranvier

Plasticity of axonal branching offers another route by which WM structure could be altered by experience, though this has not yet been demonstrated in brain WM (Figure 2G). However, there are reports of axonal branching in spinal cord WM in response to injury (Bradbury and McMahon, 2006).

Given the lack of available data on this potential plasticity mechanism in WM, it is of interest to consider the literature on gray matter as a source of inspiration for future studies of WM plasticity. Learning and experience have long been associated with dendritic and synaptic changes in gray matter though changes in axonal branching have been investigated far less extensively. *In vivo* chronic imaging of axons in the adult brain shows that, without experimental perturbation, subtypes of intracortical axonal branches can grow and retract several tens of micrometers over a few days (De Paola et al., 2006). There is some, albeit limited, evidence that cortical axons also extend or retract in response to experience (Hihara et al., 2006). For instance, a qualitative study suggested that axonal projections increase within the intraparietal sulcus in monkeys that were trained to use tools compared to controls (Hihara et al., 2006). Spatial learning induces axonal branching in the hippocampal formation (Ramírez-Amaya et al., 2001). Axonal boutons, the terminals that contact dendritic spines and where synapses are situated, can rapidly reorganize in response to experience and neuronal activity (De Paola et al., 2006, Holtmaat et al., 2008, Nikonenko et al., 2003). Future work should assess whether the experimental manipulations suggested to evoke axonal branching in gray matter produce any measurable change in WM or plasticity of long-range axonal projections.

Nodes of Ranvier, internode length, and changes in ion channel density are other features of the myelinated axon that impact conduction speed and can potentially be modulated by experience (Figures 2I and 2E). For instance, a recent study showed that changes in internode length and node of Ranvier specialization can be used to tune the arrival of action potentials along different length branches of the same axon (Ford et al., 2015). Node length has recently been shown to be more consistent within axons than between axons, and computational modeling demonstrates that variations in node length can modulate conduction speed by 20%, a similar magnitude to changes in myelin thickness or internode length (Arancibia-Cárcamo et al., 2017) (Figures 2I and 2E). This suggests that modulation of node length along the length of an axon may offer a rapid and energy-efficient means to fine-tune axon-specific conduction speed.

### Examples of White Matter Plasticity: Evidence from Human and Animal Studies

Non-invasive neuroimaging methods (Boxes 1 and 2) offer powerful approaches to study WM microstructure *in vivo*, and its change with experience, in both humans and animals. Consistent with studies in postmortem samples (Benes et al., 1994), *in vivo* imaging investigations have revealed that WM development continues well into adulthood (Giedd, 2004, Lebel and Beaulieu, 2011, Sexton et al., 2014, Sowell et al., 1999) and WM decline accelerates beyond middle age, around 50 years old (Burzynska et al., 2010, Lebel et al., 2012, Sexton et al., 2014). Beyond these age-related changes, there is growing evidence that experience results in dynamic changes in WM structure throughout the lifespan.

Box 1 **Imaging White Matter in the Human Brain**

WM structure can be studied using non-invasive brain imaging techniques. The most commonly used approaches rely on magnetic resonance imaging (MRI). For example, diffusion-weighted imaging (DWI) is sensitive to self-diffusion of water molecules in tissue and will therefore be affected by alterations in local tissue microstructure. Typically, multiple different brain images are acquired using DWI, with each one sensitized to diffusion in a different direction in space. This provides, for each brain voxel, a series of measurements describing the amount of water diffusion measured along a particular direction in space. Mathematical models can be fit to those measurements, with the complexity of the possible models depending in part on the number of directions sampled. The most commonly used model is the diffusion tensor model (DTI), in which diffusion at each voxel is described by a tensor or ellipsoid. This allows for voxel-wise estimation of several useful model parameters, such as fractional anisotropy (FA), which estimates the directional dependence of the water diffusion, or mean diffusivity (MD), which estimates the average diffusion across all directions. In WM, water diffusion is highly directionally dependent as water diffuses more easily along the axis of a fiber bundle than across it, due to physical barriers such as cell membranes or myelin. As such, FA is modulated by several features, which we will collectively refer to as “white matter microstructure” (Box 2) (Zatorre et al., 2012). For instance, high axon packing density and low axon diameter translate into high FA values in organized WM due to high membrane density perpendicular to the axon (Takahashi et al., 2002, Zatorre et al., 2012). Although myelin is not necessary for anisotropy, FA is altered when myelin is absent or damaged, with the absence of myelin decreasing FA values up to 20% in mouse models (Beaulieu and Allen, 1994, Gulani et al., 2001).

DTI is very sensitive to changes in microstructure, but it has limitations. While the tensor model is useful in regions of highly coherent fiber structure, it is more difficult to interpret in areas with fiber crossing or complexity, where there is more than one fiber direction. In addition, DTI parameters are nonspecific since there is not a direct correspondence between a DTI-derived value and a WM cellular component. Recently, more specific diffusion acquisition and analysis techniques have been developed to bypass some of the traditional DTI limitations. For instance, one method, called “Axcaliber,” uses a model of intra-axonal and extra-axonal diffusion to estimate the average axon caliber and density in a particular voxel that typically would contain thousands of axons, potentially of varying sizes (Assaf et al., 2008, Barazany et al., 2009). Other techniques, such as neurite orientation dispersion and density (NODDI) (Zhang et al., 2012) and composite hindered and restricted model of diffusion (CHARMED) (Assaf and Basser, 2005), allow for an estimation of fiber dispersion, orientation, and density.

There has been considerable effort put into developing MRI techniques that are sensitive to more specific cellular components, particularly myelin. T1-weighted imaging is a non-quantitative type of structural image that has been traditionally used to extract information about volume, density, or thickness of gray matter. Interestingly, most of the contrast in T1-weighted images is provided by myelin and iron content (Stüber et al., 2014). T1 weighted/T2 weighted ratio images can be used to calculate “myelin maps” and facilitate the identification of cortical areas based on regional differences in myelin (Glasser and Van Essen, 2011). Moreover, high-resolution quantitative T1 or 1/T1 maps reflect myeloarchitecture in cortical areas (Eickhoff et al., 2005, Sereno et al., 2013, Tardif et al., 2015). While T1 maps seem to reflect myelin content in the cortex in high-resolution images (Stüber et al., 2014), this is yet to be validated in WM.

Magnetization transfer (MT) is another MRI technique that indirectly detects water bound to macromolecules such as lipids and proteins and is thus sensitive to myelin (Kucharczyk et al., 1994). MT ratio (MTR) imaging is a semi-quantitative technique that is calculated from an MT-saturated image and a non-MT-saturated image. MTR maps are found to correlate with myelin (Barkhof et al., 2003, Schmierer et al., 2004) and have a short acquisition time at relatively high resolution. However, this method, as with any non-quantitative or semi-quantitative method, has several disadvantages, including a lack of standardization across imaging centers, making it hard to compare results (for review, see Alexander et al., 2011). Quantitative MT (qMT), on the other hand, provides a better characterization of the underlying tissue than MTR and is more sensitive and specific to myelin (Schmierer et al., 2007, Sled et al., 2004). For instance, it has been demonstrated that qMT is able to show regional differences in WM of healthy participants that are related to tissue myelination (Sled et al., 2004) and that qMT measures reflect myelination in multiple sclerosis (Schmierer et al., 2007). However, the higher specificity and sensitivity come at the expense of resolution, coverage, and scan time (Henkelman et al., 2001, Schmierer et al., 2008, Schmierer et al., 2010).

In conclusion, structural MR measures are nonspecific and multimodal approaches can provide a more detailed picture of the cellular mechanisms that underlie WM plasticity in humans. Recent developments in MRI acquisition and analysis techniques are now allowing for a more specific characterization of the underlying tissue and higher sensitivity, but more work is needed on validation and optimization of these methods. Such advances will greatly enhance our ability to interpret neuroimaging measures of WM plasticity in cellular terms (Box 3).

Box 2 **White Matter Microstructure and White Matter Integrity: What Do They Mean?**

In neuroimaging, WM microstructure is often used as a shorthand to refer to the WM features present at the microscopic level that can be indirectly measured by MR methods such as diffusion imaging. The specific features referred to will depend on the sensitivity of the method in question. For diffusion imaging, this would include, for example, axon diameter and density, fiber organization, myelin content, glial cells, and the condition or permeability of the membrane. As such, any change or group difference in a measure derived from diffusion imaging (e.g., FA, MD, radial diffusivity [RD], etc.) may reflect a change or difference in any of these underlying microstructural features. WM integrity is another commonly used term, typically used interchangeably with WM microstructure. However, as the term “integrity” (or rather its inverse) carries implications of pathology or degeneration, in this review we favor the expression “white matter microstructure” as most of the examples we will discuss are not from a pathological context.

There is extensive evidence from both humans and animal models that skill learning has an impact on WM. Environmental and social factors have mostly been investigated in animal models, and there is currently no clear evidence of their effects on WM of humans. Although less well demonstrated, lifestyle factors such as exercise and sleep have been found to correlate with WM measures and are potentially important mediators of WM plasticity during adulthood. In the following sections, we provide an overview of the main evidence for experience-dependent WM plasticity during adulthood.

#### Skilled Performance and Learning

Cross-sectional human neuroimaging studies have shown that WM microstructure correlates with skilled performance, with correlations typically localized in pathways that are functionally relevant to the skill. For instance, fractional anisotropy (FA) within a corpus callosum area that connects both motor cortices was found to correlate positively with performance in a novel bimanual task (Johansen-Berg et al., 2007). Such relationships can also be found for long-standing skills, honed after years of practice. For example, in pianists, FA within the internal capsule, which includes corticospinal tract fibers, was shown to correlate positively with the number of hours of musical practice during childhood (Bengtsson et al., 2005). Other motor activities such as dancing or typing (Cannonieri et al., 2007) have also been shown to have effects on WM. Although higher FA or WM volume has more often been associated with greater skill, some studies have found the opposite relationship. For instance, professional ballet dancers compared to controls were found to have lower gray matter, lower volume in the underlying WM, lower FA in WM areas underlying bilateral premotor cortex, and lower brain activation, which correlated with age of onset of dancing (Hänggi et al., 2010). While the functional findings fit with previous demonstrations that experts have reduced BOLD signal in brain areas associated with their expertise (Haslinger et al., 2004, Meister et al., 2005), the structural findings are more challenging to interpret. Variables such as amount of training, learning stage, or learning strategy might underlie some of the discrepancies between studies. For example, we have previously reported that amount of practice has differential effects on gray and WM structure (Sampaio-Baptista et al., 2014). In the cognitive domain, studies have demonstrated cross-sectional associations between WM microstructure and, for instance, memory (Charlton et al., 2010, Rudebeck et al., 2009), reading ability (Carreiras et al., 2009, Klingberg et al., 2000), grammar learning (Flöel et al., 2009), and mental rotation (Wolbers et al., 2006). These studies suggest that variation in WM structure may be due in part to experience-dependent plasticity. However, due to the nature of the design, cross-sectional studies are unable to distinguish between experience-dependent structural changes and pre-existing structural conditions that determine behavior and performance.

**Figure 3:**
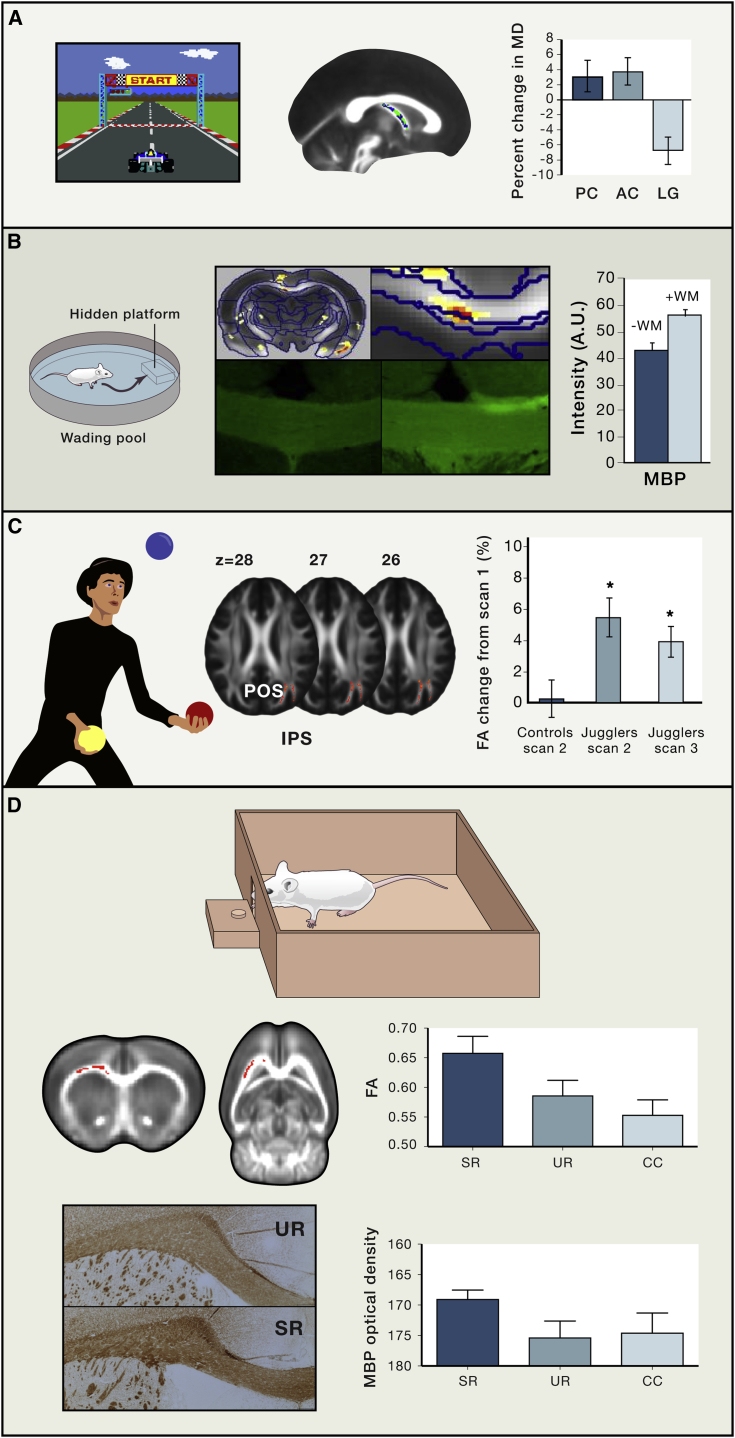
Neuroimaging Findings of White Matter Plasticity in Humans and Rodents with Spatial and Motor Skills (A) Learning a spatial navigation task results in rapid decreases in MD in the fornix (Hofstetter et al., 2013). (B) Morris water maze task acquisition results in changes in WM and higher myelination (Blumenfeld-Katzir et al., 2011). (C) Learning a new motor skill results in increases in FA (Scholz et al., 2009). (D) Skill learning results in higher FA and higher myelination (Sampaio-Baptista et al., 2013). Error bars represent SE.

Longitudinal studies, which are able to test directly for experience-driven changes in WM structure, have provided some evidence that learning can induce structural plasticity in WM in humans. For example, 6 weeks of juggling practice resulted in increases in FA in WM tracts that co-localized with increases in gray matter areas that are related to reaching and grasping movements (Scholz et al., 2009) (Figure 3). Similarly, working-memory training increased in FA in parietal fibers (Takeuchi et al., 2010) while visual perception learning increased FA in tracts underlying the visual cortex in older adults, but not younger (Yotsumoto et al., 2014). Such changes can happen remarkably quickly—for example, just 2 hr of training in a car racing game that required navigational skills resulted in immediate decreases in mean diffusivity (MD) in the fornix, a WM pathway associated with the hippocampus (Hofstetter et al., 2013) (Figure 3).

Most such studies have reported that training results in increased FA or decreased MD, a pattern typically associated with increased myelin or increased packing density of a fiber bundle. However, changes in the opposite direction have also been reported. For example, participants that trained in a whole-body balancing task showed decreases in FA and a negative correlation between FA changes and performance in prefrontal areas (Taubert et al., 2010). This highlights a challenge in interpreting measures such as FA, which do not show a straightforward relationship to the underlying fiber architecture or tissue properties. In this case, the decrease in FA was interpreted as being due to changes in areas of crossing fibers, where, for example, selective strengthening of a minor fiber population in a crossing fiber region could result in a decrease in FA (Taubert et al., 2010).

As diffusion metrics such as FA are modulated by many different WM features, such as myelin, axon diameter, and axonal density, it is not possible to pinpoint the cellular events that are changing the diffusion signal in these studies. Recently, rodent studies have used a combination of magnetic resonance imaging (MRI) techniques and histology to shed light on the cellular mechanisms of WM plasticity in learning (Blumenfeld-Katzir et al., 2011, Sampaio-Baptista et al., 2013). For example, we recently reported higher FA and myelin expression in a rat model of motor learning (Sampaio-Baptista et al., 2013) (Figure 3). Additionally, greater myelination, as shown by immunohistochemistry, was related to better performance in the task (Sampaio-Baptista et al., 2013). The apparent importance of myelin plasticity in this motor learning paradigm is consistent with a recent study using a conditional transgenic mouse model to provide causal evidence that new myelin is required for novel motor learning (McKenzie et al., 2014). New evidence suggests that oligodendrocyte plasticity might even play a role in the very first hours of skill acquisition. Preventing the differentiation of new oligodendrocytes resulted in a deficit in complex wheel running within 3 hr (Xiao et al., 2016). Additionally, maturation of new OL was found to be increased in WM within the same time frame in response to experience in the complex wheel (Xiao et al., 2016).

Beyond the motor domain, a role for myelin plasticity in learning was also found in a prior neuroimaging study that used a spatial navigation task (Blumenfeld-Katzir et al., 2011) (Figure 3). However, a correlation between FA and immunohistological measures of myelin expression could not be established in any of the above neuroimaging studies. This might be due to the complexity of the FA signal. A recent study using composite hindered and restricted model of diffusion (CLARITY) and whole-brain immunolabeling along with the diffusion tensor model (DTI) demonstrated that myelin basic protein (MBP) correlates with FA in WM regions with coherent fiber orientations and low fiber dispersion, but not in areas of crossing or complex fiber architecture (Chang et al., 2017). It is possible that myelin is but one aspect of the WM that has undergone plasticity and FA is capturing the sum of all changes in WM microstructure. Further studies using other techniques, such as electron microscopy, could potentially clarify if other events such as axonal diameter changes (Figure 2H) are also contributing to FA changes (see Box 3 for discussion).

Box 3 **What Cellular Changes Are Most Likely to Underlie Neuroimaging Findings of White Matter Plasticity?**

When speculating about the cellular changes that are most likely to underlie neuroimaging findings of WM plasticity, a number of questions need to be considered, including the following: What tissue properties are the neuroimaging metrics in question sensitive to (Box 1)? What cellular changes are plausible consequences of the experimental manipulation being studied? What are the likely cellular contents of our imaging voxels?

In response to this last question, a recent report attempted to estimate, using confocal imaging of immunofluorescent markers of cell bodies and electron microscopy, the composition of such a volume (Walhovd et al., 2014). The authors estimated that a volume of 100 × 100 × 100 μm of rodent WM contains between 1,000 and 13,000 axons, plus an average of 86 oligodendrocytes, 22 astrocytes, 6.5 OPCs, and 9.5 microglia; astrocytes’ processes and oligodendrocytes were found to each cover 48% of the assessed area. The authors extrapolated that a 2 × 2 × 2 mm voxel, commonly used in human diffusion imaging studies, and assuming similar axon density in humans and rodents, would contain between 0.5 and 5 million axons, 700,000 oligodendrocytes, 185,000 astrocytes, 52,000 OPCs, and 76,000 microglia. The authors acknowledge, however, that these estimates should be considered with caution since there is not a linear relation to total brain volume and little is known about how these cell sizes and densities scale across species. For instance, it has been reported that human astrocytes are two times bigger and occupy a larger volume than those in rodents (Herculano-Houzel, 2014). Thus astrocytes (Figure 2D), along with myelin and oligodendrocytes, would be expected to make a large contribution to any voxel measurements in humans (Walhovd et al., 2014).

Considering the few studies that have combined WM MRI and histological measures in relation to learning and experience, myelination has been consistently been found to be involved (Blumenfeld-Katzir et al., 2011, Sampaio-Baptista et al., 2013), though few alternative cellular changes have been quantified for comparison. These studies are unable to distinguish between *de novo* myelination and myelin remodeling by preexisting oligodendrocytes. Considering the small number of OPCs present in each voxel, it is more likely that the main contribution to the FA changes is related to preexisting oligodendrocyte remodeling, such as change in internode length and/or thickness. Although *de novo* myelination from recently differentiated OPCs has been found to be important for motor acquisition (McKenzie et al., 2014, Xiao et al., 2016), it is likely to have a small contribution to the neuroimaging findings in human studies. Future studies using the same transgenic strains have the potential to clarify and estimate the real contribution of *de novo* myelination to the MRI measures. Timescale is a crucial factor in assessing the contributions of each myelin-related mechanism. For instance, with long timescales, such as weeks or months, the potential contribution of *de novo* myelination to the MRI signal is longer, as more time would be available for OPC proliferation, differentiation, and myelination. Additionally, changes in nodes of Ranvier size are functionally important (Arancibia-Cárcamo et al., 2017), but studies estimating their impact on neuroimaging measures are lacking.

As mentioned above, astrocytes’ contribution to the overall signal is likely to be significant given the large volume they occupy (Walhovd et al., 2014). There are few neuroimaging studies that have assessed DTI changes in relation to changes in astrocytes, and they are mainly in gray matter (Blumenfeld-Katzir et al., 2011, Johansen-Berg et al., 2012, Sagi et al., 2012). Future studies of WM plasticity should consider assessing the contribution of astrocytes relative to axons and myelin, and assessing astrocyte function in the context of learning and experience.

#### Environmental and Social Factors

Social and physical environments affect WM structure not only during development, but also in adulthood, though evidence of exactly what properties of the WM are altered by these environmental effects is mixed. For instance, 4-month-old rats (roughly equivalent in age to human young adults) housed in an enriched environment for 2 months were found to have a higher area of unmyelinated axons and glial cells in the corpus callosum (Markham et al., 2009). This study suggests that myelination may be less sensitive to experience in adulthood. However, a different study reported an increase in volume of myelinated fibers and myelin sheaths in the corpus callosum with 4 months of environmental enrichment in 14-month-old rats (equivalent in age to human older adults) (Zhao et al., 2012). The discrepancy between the studies might be due to differences in the age of the animals as well as to the duration of enrichment exposure.

Social experience seems to play a particularly important role in myelination of the prefrontal cortex. For example, studies in mice suggest there is a critical period for social experience to induce normal prefrontal cortex myelination very early in life (Makinodan et al., 2012). However, later experience can then alter prefrontal myelin: 8 weeks of social isolation in adulthood was found to decrease myelin thickness in the gray matter of mouse prefrontal cortex but had no effects in the corpus callosum (Liu et al., 2012).

#### Lifestyle Factors: Exercise and Sleep

Our lifestyle choices are increasingly recognized to influence our cognitive health throughout life (Peel et al., 2005). Consistent with this, there is growing evidence of associations between lifestyle factors and measures of brain structure and function (Erickson et al., 2014, Jackson et al., 2016). Aerobic exercise has been extensively reported to have an impact on gray matter volume of the hippocampus as measured by neuroimaging (Erickson et al., 2011, Thomas et al., 2016), but the underlying cellular drivers for gross changes following exercise interventions detected using neuroimaging remain unclear. Animal studies provide consistent evidence that exercise increases neurogenesis and angiogenesis within the hippocampus (van Praag et al., 1999a, van Praag et al., 1999b). One human study demonstrates increased blood volume in the same region after an exercise intervention (Pereira et al., 2007). But another study reported that an increase in hippocampal volume was more consistent with an increase in myelin than with an increase in vasculature in an exercise study using a range of multi-modal imaging metrics (Thomas et al., 2016).

There are also a few reports of associations between physical activity and WM structure. For instance, cross-sectional studies suggest a correlation between WM structure and amount of physical activity in older adults (Burzynska et al., 2014, Tian et al., 2015). A recent meta-analysis of 29 studies reported a small but significant positive association between physical activity and measures of WM structure (Sexton et al., 2016). The potential relevance of this association is highlighted by a recent study reporting that WM microstructure mediates the relationship between fitness and cognitive performance in older adults (Oberlin et al., 2016). In this study, a mediation analysis showed that fitness levels were associated with higher FA in WM, which, in turn, may help to preserve spatial memory performance in older adulthood (Oberlin et al., 2016). There is also some longitudinal evidence that 6 months of exercise results in increases in the volume of the anterior corpus callosum in elderly adults (Colcombe et al., 2006), while a 1 year intervention didn’t result in microstructural changes in WM (Voss et al., 2013). Rodent studies suggest that running promotes OPC proliferation and differentiation in gray and WM regions (Ehninger et al., 2011, Matsumoto et al., 2011, McKenzie et al., 2014, Simon et al., 2011), which might partially contribute to changes in WM or myelin measures seen in humans.

Another lifestyle factor that may influence cognitive and brain measures is sleep. Sleep is present across animal species and is thought to be necessary for neural network homeostasis (Tononi and Cirelli, 2003), learning, and memory consolidation (Maquet, 2001). It is well established that sleep is important for motor consolidation in humans (Fischer et al., 2002, Walker et al., 2002) and rodents (Gulati et al., 2014, Hanlon et al., 2009) and that slow waves during sleep reflect the recent neuronal activation and synaptic plasticity (Tononi and Cirelli, 2006). A recent study has found a correlation between WM variation and individual sleep oscillations in healthy individuals (Piantoni et al., 2013), suggesting that the sleep oscillations could be related not only to local cortical networks, but also to long-range structural connectivity.

There is recent evidence that the awake-sleep cycle might play a role in WM maintenance (Bellesi et al., 2013). Some cross-sectional human studies have reported that insomnia is associated with lower FA in WM regions (Li et al., 2016, Spiegelhalder et al., 2014). Rodent studies show that OPC proliferation increases during sleeping hours while differentiation is higher during the awake period (Bellesi et al., 2013). It is possible that abnormal sleeping patterns interrupt the proliferation phase of the OPCs that might play a role in myelin maintenance. However, cross-sectional studies cannot clarify if lower FA is caused by abnormal sleeping patterns or vice versa. Longitudinal studies are necessary to investigate if an improvement in sleeping patterns, for instance through pharmacological interventions, can result in a positive change in WM structure.

### Conclusions

These are exciting times for the field of WM plasticity, with enormous opportunity for fundamental new discovery. There is mounting evidence that WM change plays an important and previously neglected role in learning and plasticity throughout life. Recent years have seen particular advances in our understanding of activity-dependent myelination. Progress has been accelerated in part through methodological advances. These include methods for manipulating brain circuits, for example through optogenetic stimulation; techniques for interfering with key cellular processes, such as transgenic or pharmacological manipulation; and new approaches for monitoring WM, like *in vivo* imaging in animal models as well as in living humans.

Such rapid advances in our understanding give rise to scores of fundamental unanswered questions. For example, is activity along an axon sufficient to drive myelin plasticity or do myelin changes depend on “learning” (i.e., plasticity at the synapse) occurring? If the latter, then what signals allow for synaptic plasticity to be communicated to the oligodendrocytes? Do particular types of activity preferentially drive myelin plasticity? To what extent do changes in other features of the myelinated axon, such as axon diameter or node length, alter with experience or learning? Are local changes in myelin used to regulate signal timing across distributed circuits? If so, then what signals communicate effective synchronization back to the oligodendrocyte? Can activity-dependent process decrease, as well as increase, myelination?

Addressing these questions requires investigations that span across scales. Continued technological advances will help to fuel these investigations. For example, advances in automated methods for volumetric reconstruction of tissue at ultrastructural resolution (Briggman and Bock, 2012), alongside efforts to build models to translate between MRI signals and microscopy (Chen et al., 2013, Stüber et al., 2014), will help both to push the boundaries of investigation and to relate evidence across scale. Such cross-scale opportunities bring us closer to bridging the gap between fine-grained molecular mechanisms of WM plasticity and understanding the role it plays in complex behavior and learning.
